# Immune podocyte injury in autoimmune glomerular diseases

**DOI:** 10.3389/fimmu.2026.1804416

**Published:** 2026-03-19

**Authors:** Han Zhu, Jianing Sun, Yan Yan, Peng Liu, Yong Huang

**Affiliations:** 1 Jiangxi University of Chinese Medicine, Nanchang, Jiangxi, China; 2 Xiyuan Hospital, China Academy of Chinese Medical Sciences, Beijing, China; 3 Department of Nephrology, Affiliated Hospital of Jiangxi University of Traditional Chinese Medicine, Nanchang, Jiangxi, China

**Keywords:** autoimmune glomerular diseases, complement activation, pathogenesis, podocyte immune injury, protein uric kidney disease

## Abstract

Autoimmune glomerular diseases (AGDs) are immune dysregulation-driven disorders of the glomerulus and a major cause of chronic kidney disease (CKD) and end-stage kidney disease (ESKD). The glomerular filtration barrier, formed by fenestrated endothelial cells, podocytes, and the glomerular basement membrane (GBM), is indispensable for renal homeostasis. Podocytes are terminally differentiated epithelial cells and are difficult to replace once injured. In susceptible individuals, maladaptive activation of humoral and cellular immunity promotes autoantibody formation, immune complex deposition, and complement activation within the glomerulus, leading to early proteinuria and progressive loss of kidney function. Across clinically heterogeneous AGDs, podocytes represent a key convergence point at which immune effector signals are translated into structural and functional barrier failure. Accumulating evidence further suggests that podocytes are not merely passive targets but active “immune podocytes” capable of engaging innate danger-sensing pathways and adopting adaptive immune-like programs that shape local inflammation and disease evolution. This Review synthesizes current advances in immune-mediated podocyte injury, with emphasis on complement-linked signaling, podocyte-intrinsic inflammatory circuits, and podocyte-associated immune interactions. We relate these mechanisms to cytoskeletal remodeling, organelle stress, and regulated cell-death pathways that culminate in podocyte depletion and glomerulosclerosis. We also discuss podocyte protective responses and emerging opportunities for precision, podocyte-centered therapeutics to improve long-term outcomes in AGDs.

## 1 Introduction

Autoimmune glomerular diseases (AGDs) are immune dysregulation-driven disorders of the glomerulus and a major cause of end-stage kidney disease (ESKD). This umbrella term encompasses clinically and mechanistically diverse entities, including membranous nephropathy (MN), minimal change disease (MCD), focal segmental glomerulosclerosis (FSGS), IgA nephropathy (IgAN), and lupus nephritis (LN) (1). A defining feature of these disorders is that, in genetically susceptible individuals, aberrant recognition of self-antigens inappropriately activates T lymphocytes (T cells) and B lymphocytes (B cells), promoting autoantibody production. The ensuing immune complexes deposit in the glomerulus, amplify injury through complement activation, and sustain local inflammation, ultimately causing glomerular cellular damage and architectural disruption (2).

**Figure 1:**
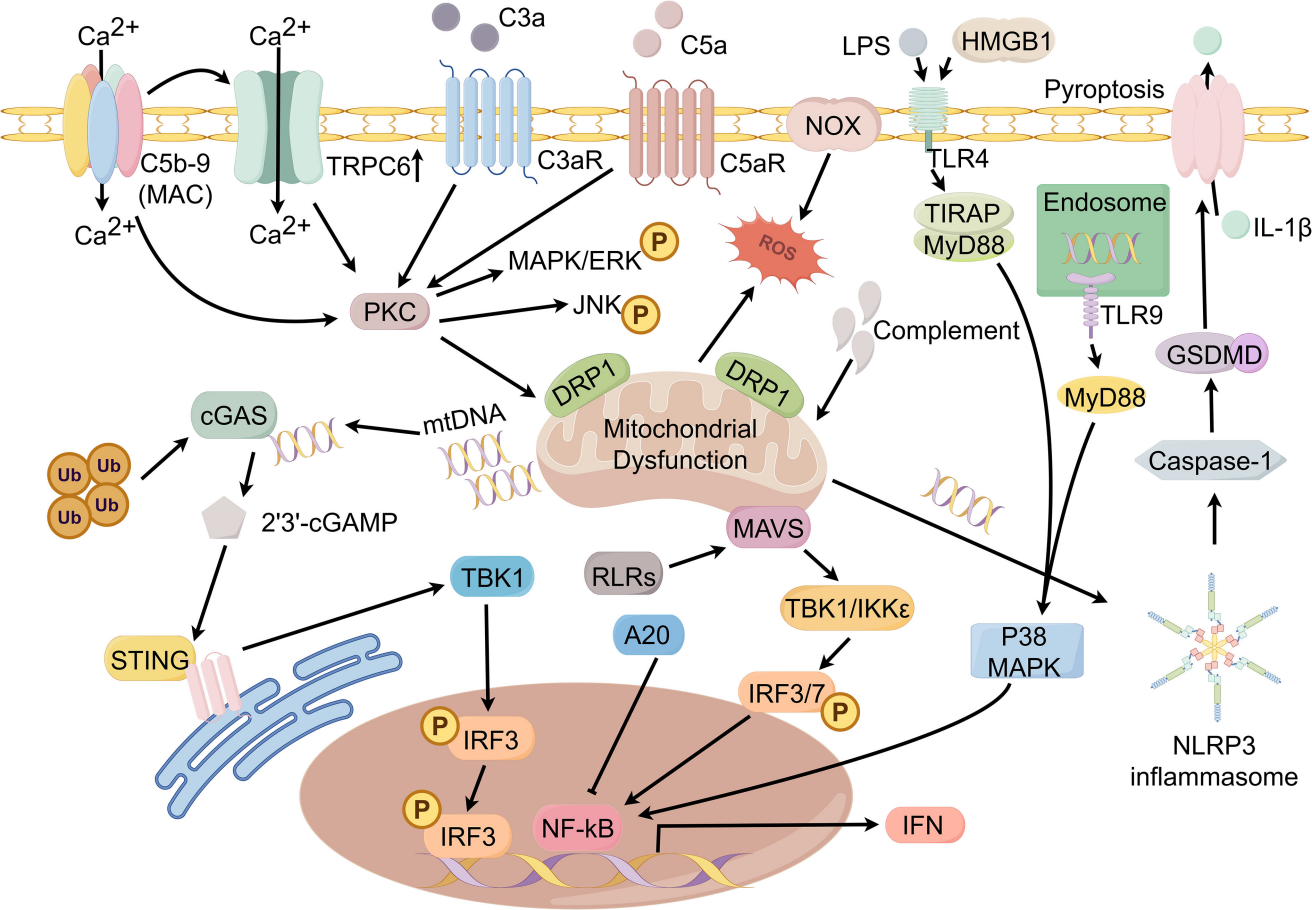
Innate immune sensing and damage signaling networks in podocytes.

**Figure 2:**
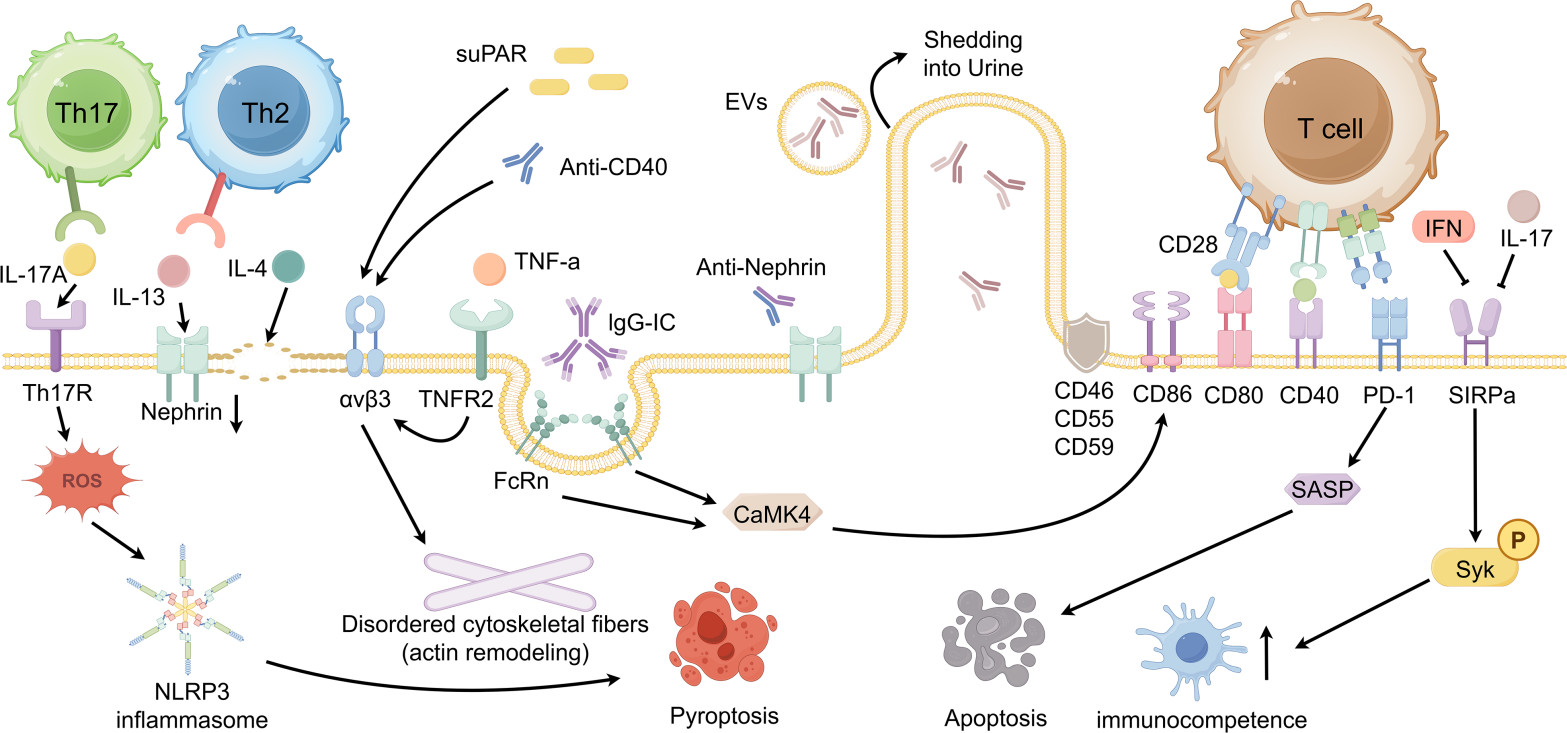
The podocyte-adaptive immune landscape: crosstalk and responses.

Podocytes are often one of the earliest and most vulnerable cell targets in immune-mediated glomerular injury. Existing research evidence shows that podocytes are not only passive damage targets, but also active participants in glomerular local immune regulation. Podocytes can express immune-related molecules, including components of antigen presentation mechanisms and complement regulators, thereby participating in innate and adaptive immune responses. Therefore, podocytes are called “immune podocytes” in the glomerular microenvironment (3). Podocyte immune dysfunction is closely related to the pathogenesis of various AGD. Although some progress has been made in recent years, the mechanism by which the immune response damages podocytes is still not completely clear. Delineating these pathways is crucial for the development of accurate podocyte-targeted therapy. In this review, we focus on immune complex deposition and complement activation, podocyte innate immune signaling, and podocyte-associated adaptive immunity (**Figure 1** and **Figure 2**, made by Figdraw). We combined recent findings to link these molecular pathways to podocyte injury.

## 2 Podocyte injury and underlying mechanisms

Immune complex deposition and complement activation are common upstream mechanisms in AGDs. However, the principal immune triggers and podocyte response programs are disease-specific and should be stated explicitly to enable clinical translation. In primary membranous nephropathy (PMN), podocyte-targeted autoimmunity together with downstream complement effector pathways establishes a relatively direct link from biomarkers to targeted therapy. In LN, immune complex-driven inflammation converges with podocyte intrinsic stress programs, including complement-associated mitochondrial injury and interferon-related signatures, that may not be reflected by proteinuria alone. By contrast, primary podocytopathies such as MCD and FSGS emphasize cytokine and costimulatory pathways such as CD80, also termed B7-1, and proposed circulating mediators such as soluble urokinase-type plasminogen activator receptor (suPAR) for which clinical support varies across cohorts, necessitating cautious interpretation. In the following sections, we therefore connect these disease-biased pathways to candidate serologic and urinary readouts, including emerging autoantibody signals and urinary extracellular vesicle biomarkers, to inform therapeutic stratification and response monitoring.

### 2.1 Immune complex deposition and complement activation

In autoimmune-mediated glomerular injury, podocytes often become a direct target of humoral immune attacks. The current popular view is that antigen-specific antibodies bind to autoantigens on the surface of podocytes and form *in situ* immune complexes along the glomerular basement membrane (GBM), thereby initiating complement activation. This process eventually leads to the membrane attack complex (MAC; c5b-9) being deposited at the GBM interface of podocytes, directly impairing the structural integrity of the glomerular filtration barrier (4). A variety of podocyte antigens and their corresponding autoantibodies have been identified, among which phospholipase A2 receptor (PLA2R) and type 1 thrombospondin domain containing 7A (THSD7A) are the most characteristic. Anti-PLA2R antibody and anti-THSD7A antibody are detected in about 50% -80% and 3% -5% of patients with PMN, respectively, and are associated with podocyte injury (5). It is important to note that the molecular consequences of sublethal complement attacks are not limited to cell membrane perturbations, but also involve complex intracellular signaling cascades. In podocytes, C5b-9 can induce Ca^2+^ influx and activate protein kinase C (PKC), mitogen-activated protein kinase/extracellular signal-regulated kinase (MAPK/ERK), and c-Jun N-terminal kinase (JNK) pathways. At the same time, the increase of NADPH oxidase activity leads to the excessive production of reactive oxygen species (ROS), which causes oxidative stress and destroys the highly organized actin cytoskeleton supporting the structure and function of podocytes (6, 7).

Interestingly, the predominance of immunoglobulin G4 (IgG4) in PMN does not mean that complement activation is confined to passive engagement of the classical pathway. In MN, aberrant Fc glycosylation of anti-PLA2R IgG4 may facilitate mannose-binding lectin (MBL) binding and lectin pathway activation, providing a mechanistic rationale for complement deposition despite IgG4 predominance (8). Notably, recent studies indicate that complement activation can also subvert podocyte homeostatic programs. Sublytic C5b-9 upregulates transient receptor potential cation channel subfamily C member 6 (TRPC6) and suppresses autophagic flux via Ca^2+^-extracellular signal-regulated kinase 1/2 (ERK1/2) signaling (9). In LN, C5a-C5a receptor 1 (C5aR1) engagement promotes dynamin-related protein 1 (DRP1)-dependent mitochondrial dysfunction and ROS generation (10). Together, these findings position complement as both a cytolytic effector and a signaling driver of podocyte injury, underscoring its appeal as a target for pathway-directed intervention.

Beyond the MAC, complement-derived fragments can also directly injure podocytes. The anaphylatoxin C3a binds the C3a receptor (C3aR) on the podocyte surface and initiates pathogenic signaling. In PMN, the C3a-C3aR axis has emerged as a key effector of complement-mediated podocyte injury: glomerular C3aR expression is markedly increased and correlates with proteinuria severity. *In vitro*, C3a impairs podocyte function, and pharmacological C3aR blockade attenuates podocyte injury. Consistent with these observations, C3aR antagonism in animal models significantly ameliorates proteinuria and mitigates podocyte structural damage (11).

Recent work further suggests that sublytic complement attack can engage regulated cell-death programs in podocytes. In experimental MN, complement-induced mitochondrial dysfunction and ROS accumulation activate the NLR family pyrin domain-containing 3 (NLRP3) inflammasome and gasdermin D (GSDMD), thereby promoting pyroptotic podocyte death and exacerbating injury (12).In FSGS, reduced glutathione peroxidase 4 (GPX4) expression, together with lipid peroxidation signatures, implicates ferroptosis. Moreover, pharmacological ferroptosis inhibition ameliorates proteinuria, highlighting iron-dependent lipid peroxidation as a therapeutically actionable pathway to podocyte loss (13).

Overall, humoral immunity-driven immune complex deposition and complement activation represent central mechanisms of podocyte injury in autoimmune nephritides and key drivers of nephrotic syndrome initiation and progression.

### 2.2 Podocyte-intrinsic innate immunity

Podocytes are not merely passive targets of immune assault; they also exhibit innate immune capacities that parallel selected functions of professional immune cells. Podocytes express pattern-recognition receptors (PRRs), including Toll-like receptors (TLRs) and nucleotide-binding oligomerization domain (NOD)-like receptors (NLRs), enabling responses to pathogen- and danger-associated signals (14, 15). For example, the Toll-like receptor 4 (TLR4) expressed by podocytes recognizes bacterial lipopolysaccharide (LPS). Under high glucose stress, the TLR4 signal can be abnormally amplified, which aggravates podocyte injury and accelerates renal fibrosis (16). Similarly, aldosterone-induced podocyte injury depends on the activation of NLRP3 inflammasome, and silencing NLRP3 can significantly reduce the resulting damage (17). In LN, NLRP3 activation in podocytes can be directly driven by immune-derived IgG rather than metabolic stress alone. Fu et al. showed that lupus serum activates podocyte NLRP3 via mitochondrial ROS, and IgG depletion abrogated caspase-1 activation; pharmacologic NLRP3 inhibition ameliorated proteinuria and attenuated foot-process effacement in lupus-prone mice. This research supports an LN-biased IgG-mitochondrial stress-NLRP3 axis (18).And studies have found endogenous mitochondrial DNA can activate TLR9 to participate in nuclear factor kappa B (NF-κB) and p38 mitogen-activated protein kinase (p38 MAPK) signal transduction and promote apoptosis. In autoimmune nephritis models, increased podocyte TLR9 activity is associated with more severe glomerular lesions (19). Crosstalk with macrophage-derived mediators such as tumor necrosis factor alpha (TNF-α) and interleukin 1 beta (IL-1β) may further amplify intraglomerular inflammation, whereas activation of the NLRP3 inflammasome represents an additional convergent node of injury (20).

It is worth noting that the nucleic acid-sensing/interferon axis is increasingly viewed as an upstream innate immune driver in AGDs. Recent studies have highlighted a pivotal role for cyclic GMP-AMP synthase (cGAS) and its adaptor, stimulator of interferon genes (STING), in podocyte innate immunity. Upon sensing aberrant cytosolic DNA, podocyte cGAS catalyzes production of the second messenger 2′3′-cyclic GMP-AMP (2′3′-cGAMP), thereby activating the STING-TANK-binding kinase 1 (TBK1)-interferon regulatory factor 3 (IRF3) axis and inducing antiviral inflammatory mediators, including type I interferons (IFNs). In LN, studies further indicate that specific ubiquitination regulators can potentiate cGAS-STING-type I interferon (IFN-I) signaling in podocytes, thereby exacerbating glomerular injury (21).

And retinoic acid-inducible gene I-like receptor (RLR) signaling provides mechanistic clues to how podocytes undergo phenotypic drift under chronic immune stimulation. RLRs—including retinoic acid–inducible gene I (RIG-I), melanoma differentiation–associated protein 5 (MDA5), and laboratory of genetics and physiology 2 (LGP2)—detect exogenous RNA or aberrant self RNA and, through coupling to mitochondrial antiviral-signaling protein (MAVS), activate TBK1/inhibitor of nuclear factor kappa-B kinase subunit epsilon (IKKϵ) and interferon regulatory factors 3/7 (IRF3/7). This activation drives transcription of IFNs and proinflammatory mediators. Under immune dysregulation, the canonical antiviral module may be repurposed into a self-sustaining amplifier of inflammation (22). Studies indicate that poly(I: C)—a double-stranded RNA mimetic that activates Toll-like receptor 3 (TLR3) and RLRs—upregulates RIG-I and MDA5 in podocytes, with concomitant increases in interferon beta (IFN-β) and interleukin 6 (IL-6) expression. These responses are accompanied by actin cytoskeletal remodeling, suggesting that nucleic acid–sensing pathways can directly perturb podocyte structural homeostasis and compromise barrier function (23). Consistently, gain-of-function variants in DDX58, which encodes RIG-I, reported in lupus nephritis support a pathogenic contribution of hyperactive RLR-interferon signaling to autoimmune kidney injury. Notably, treatment with the Janus kinase (JAK) inhibitor baricitinib was associated with suppression of interferon signaling in the affected individual, suggesting that disruption of the interferon-JAK-signal transducer and activator of transcription (STAT) feed-forward loop may indirectly dampen the chronically heightened innate immune state of glomerular resident cells, including podocytes, under sustained interferon exposure (24).

In parallel, injured podocytes can release the danger-associated molecular pattern high-mobility group box 1 (HMGB1). HMGB1 can engage the receptor for advanced glycation end products (RAGE) and TLR4 on the podocyte surface, thereby promoting injurious programs, including regulated cell death and phenotypic transition (25). Available evidence indicates that interrupting HMGB1-driven signaling alleviates podocyte apoptosis and functional impairment, supporting HMGB1 as a promising therapeutic target (26).

In general, these studies have shown that the innate immune pathway of podocytes plays a dual role in autoimmune kidney disease: it contributes to pathogen defense, but when over-activated or abnormally activated, it also promotes autonomous damage of podocytes.

### 2.3 Podocyte-associated adaptive immunity

The adaptive immune response also plays an important role in podocyte injury. In addition to being a target for immune attack, podocytes also have an antigen-presenting cell (APC) -like function. Podocytes express major histocompatibility complex (MHC) class I and II molecules and can induce up-regulation of costimulatory ligands for antigen presentation and activation of T cells under inflammatory conditions (27). Further studies have shown that podocytes can induce the expression of costimulatory molecule CD40, which binds to the CD40 ligand (CD40L) on CD4^+^ T lymphocytes. This interaction can promote T cell activation and amplify local inflammatory damage (28).In the inflammatory state, podocytes can abnormally express costimulatory molecule B7-1 (CD80). By binding to CD28 on T cells, CD80 can transmit the required second signal, thereby amplifying the activation of T cells. Abnormal expression of CD80 in podocytes was detected in nephrotic syndrome with podocyte lesions, such as MCD, in the active stage of the disease (29). Podocytes also can take up immune complexes containing immunoglobulin G (IgG) through the newborn Fc receptor (FcRn), thereby promoting antigen processing and cross-presentation (30). It has been reported that FcRn-mediated IgG uptake in podocytes triggers abnormal activation of calcium and calmodulin-dependent protein kinase IV (CaMK4) (31).In podocytes, increased CaMK4 expression is associated with upregulation of CD86, thereby enhancing costimulatory signals to T cells. This shift will establish a self-enhanced immune circuit, further exacerbating local inflammation and tissue damage (32). Taken together, these studies suggest that non-traditional immune crosstalk between T cells and podocytes may exacerbate glomerular inflammatory injury.

Whether podocytes act as bona fide APC *in vivo* in human autoimmune glomerular diseases remains actively debated, and the strength of supporting evidence varies across experimental systems and disease contexts. Much of the current literature derives from *in vitro* studies and animal models showing inflammation-inducible MHC class II expression and antigen handling by podocytes. However, these findings do not establish podocytes as dominant APC within human glomeruli. Moreover, molecules frequently invoked in this discussion, including CD80, also termed B7-1, may represent broader injury-activated programs rather than a discrete APC phenotype. Accordingly, their disease-specific diagnostic and therapeutic value remains controversial in clinical studies (33). We therefore frame podocyte immune-like features as context-dependent amplifiers of local inflammation and immune crosstalk, while recognizing that clinical translation will require standardized biomarkers and rigorous validation in human studies.

In addition to the classical MHC-costimulatory axis and Fc receptor-mediated pathways, new evidence suggests that podocytes can express immune checkpoint-related molecules, thereby affecting cell outcomes. In particular, programmed cell death protein 1 (PD-1) signaling has been reported to be up-regulated in the context of glomerular disease, and its activation is associated with podocyte loss (34). Experimental data show that up-regulation of PD-1 promotes the expression of senescence-associated secretory phenotype (SASP) and podocyte apoptosis, reduces resistance to chronic inflammation and immune stress, and accelerates proteinuria and glomerulosclerosis. PD-1 blockade attenuates proteinuria in experimental FSGS, suggesting that immune checkpoint pathways may be therapeutically targeted in podocytes, not only in immune cells. Similarly, the signaling regulatory protein alpha (SIRPα) expressed by podocytes acts as an inhibitory checkpoint to inhibit immune activation by inhibiting the phosphorylation of spleen tyrosine kinase (Syk) and limiting the antigen presentation ability of podocytes. However, elevated type I and type II interferons and inflammatory mediators such as IL-17 significantly down-regulated the expression of SIRPα in podocytes. This decrease in turn relieves the inhibition of Syk signaling and enhances the immunostimulatory activity of podocytes (35). This mechanism may help to explain the enhanced ability of podocytes to promote T cell activation under high inflammatory conditions, and underscores the decisive role of the inflammatory environment in shaping the immune phenotype of podocytes.

T cell disequilibrium and T cell-derived cytokines are widely regarded as major drivers of podocyte dysfunction, and accumulating evidence suggests that T helper 17 (Th17)-associated mediators exert distinctive injurious effects on podocytes. Interleukin 17A (IL-17A) produced by Th17 cells can directly injure podocytes by promoting actin cytoskeletal remodeling, oxidative stress, apoptosis, and NLRP3 inflammasome-dependent IL-1β maturation and release (36, 37). Clinically, expansion of Th17 cells and elevated interleukin 17 (IL-17) levels are associated with more severe podocyte injury and greater proteinuria (38). Some studies further propose that activation of the NLRP3 inflammasome in podocytes not only exacerbates podocyte structural and functional impairment but may also, through cytokine networks, influence T cell differentiation and skew helper T cells toward a proinflammatory Th17 phenotype. This shift could, in turn, foster a microenvironment that sustains a self-perpetuating inflammatory loop (39). By contrast, Th2 cytokines, particularly interleukin 13 (IL-13), have been implicated in the pathogenesis of MCD. Excess IL-13 can reprogram podocyte transcriptional programs and markedly downregulate slit diaphragm proteins such as nephrin, a key slit diaphragm protein, thereby promoting foot process effacement and increasing glomerular filtration barrier permeability (40). Clinical observations likewise indicate that IL-13 levels are frequently elevated in patients with MCD. This Th2-skewed milieu may promote B cell activation and autoantibody production, thereby further aggravating podocyte injury (41).

Concurrently, B-cell-mediated humoral immunity contributes to adaptive mechanisms of podocyte injury (42). Reports have identified circulating autoantibodies against nephrin in a subset of patients with FSGS (43).In recurrent FSGS after kidney transplantation, anti-nephrin antibodies have been reported to induce nephrin phosphorylation and mislocalization in graft podocytes, accompanied by foot process effacement and severe proteinuria. Together, these observations suggest that anti-nephrin autoantibodies may act as pathogenic mediators of podocyte injury (44).In addition, certain B-cell-derived cytokines can act directly on podocytes. For example, B-cell-derived interleukin 4 (IL-4) has been reported to act on podocytes, inducing foot process effacement and proteinuria (45).

In addition to the mechanisms described above, autoimmunity- and inflammation-related factors can directly contribute to podocyte injury. suPAR has been proposed as a circulating pathogenic mediator in FSGS. In some cohorts, circulating suPAR levels are reportedly elevated, and suPAR can activate podocyte αvβ3 integrin, thereby driving cytoskeletal reorganization and foot process effacement (46). Circulating suPAR is influenced by systemic inflammation and comorbid conditions. In addition, heterogeneity in suPAR isoforms and measurement platforms has likely contributed to inconsistent associations across cohorts. These limitations warrant cautious interpretation when suPAR is considered a disease-specific biomarker. Studies further suggest that the pathogenicity of suPAR may depend on specific isoforms or synergistic interactions with other factors. For example, *in vivo*, the proteinuria-inducing effect of anti-CD40 antibodies is markedly potentiated in the setting of elevated suPAR, and the two appear to cooperate in activating podocyte αvβ3 integrin (47). Similarly, systemic inflammatory mediators such as TNF-α can act directly on podocytes. Through tumor necrosis factor receptor 2 (TNFR2), TNF-α activates intracellular signaling that promotes excessive αvβ3 integrin activation and actin filament remodeling, thereby driving foot process effacement and proteinuria (48).

Collectively, evidence indicates that in AGDs, inflammatory cues can reprogram podocytes toward APC-like and checkpoint-shifted states that reinforce T-cell and humoral responses, thereby amplifying injury and proteinuria.

### 2.4 Podocyte adaptive responses

While actively participating in immune responses, podocytes also deploy protective adaptive programs to withstand sustained immune stress. One such strategy is the induction of endogenous negative regulators that constrain excessive amplification of inflammatory signaling. Among these, A20/tumor necrosis factor alpha-induced protein 3 (A20/TNFAIP3) is a pivotal negative regulator of the NF-κB pathway. In podocytes, it is rapidly upregulated following TLRs stimulation and related cues, thereby limiting overproduction of proinflammatory cytokines (49). The role of A20 in maintaining glomerular homeostasis and restraining inflammatory responses is supported by experimental evidence. Podocyte-specific A20 deletion in mice leads to spontaneous, severe proteinuria and podocyte loss, accompanied by pronounced macrophage infiltration and heightened inflammation. Conversely, A20 expression in intact podocytes suppresses inflammatory chemokine expression and limits pathological immune cell recruitment, thereby protecting podocytes from excessive inflammatory damage during renal injury (50).

Second, podocytes express complement regulatory proteins that curb excessive complement activation. For example, podocytes secrete soluble complement factor H and express multiple membrane-associated regulators, including membrane cofactor protein (CD46), decay-accelerating factor (CD55), and protectin (CD59). Together, these regulators act in concert to prevent uncontrolled complement amplification at the podocyte surface (51, 52). Third, podocytes can enhance autophagic activity and actin-dependent endocytosis to clear deposited immune complexes as well as cellular components damaged by complement and oxidative stress. By removing these injurious stimuli, podocytes can mitigate self-injury and preserve intracellular homeostasis (53, 54). These adaptive responses support podocyte survival and function, conferring a degree of resilience in the context of inflammatory kidney injury.

Podocytes may also possess an active mechanism for handling immune complexes. Recent reports indicate that podocytes can shed membrane-associated molecular aggregates bearing self-antigens and bound antibodies as extracellular vesicles into the urine. These “autoantibody-triggered extracellular vesicles” may facilitate, at least in part, clearance of immune complexes from the podocyte surface. However, excessive vesicle release may concomitantly deplete key foot process proteins, thereby promoting widespread foot process effacement and podocyte dysfunction (55).

## 3 Conclusions and perspectives

Podocytes are pivotal determinants of glomerular integrity in AGDs, and their injury constitutes a common pathway leading to proteinuria and progressive kidney failure. While immune complex deposition and complement activation have long been regarded as principal effectors of glomerular injury, accumulating evidence suggests that podocytes are not merely passive targets but active “immune podocytes” that couple innate danger sensing with adaptive immune-like programs, thereby shaping local inflammation and disease progression. These immune modules converge on cytoskeletal remodeling, mitochondrial and metabolic stress, and regulated cell-death pathways, thereby influencing whether injury is reversible or culminates in podocyte loss and glomerulosclerosis. Building on these mechanistic insights, an expanding portfolio of pathway-specific targets is emerging for immune-mediated podocyte injury, encompassing inflammasome and nucleic acid-sensing pathways, HMGB1-RAGE/TLR signaling, and cytokine and costimulatory axes (56).

As podocyte-centered therapies move into a more translational phase, linking disease-relevant immune axes to actionable biomarkers will be essential for therapeutic decision-making and for reducing reliance on broad immunosuppression. In antibody-driven diseases such as MN, serial assessment of podocyte antigen-directed autoimmunity together with complement effector activity offers a coherent framework for monitoring immunologic activity and for guiding treatment escalation or de-escalation. In immune complex-mediated diseases such as LN, emerging podocyte stress-pathway signatures, including complement-associated mitochondrial injury modules and extracellular vesicle-based liquid biopsy readouts, may complement conventional endpoints beyond proteinuria. Future podocyte-centered trials will increasingly incorporate biomarker panels as enrichment strategies and pharmacodynamic endpoints, thereby accelerating the transition from descriptive pathology to precision immunotherapy. For example, blocking pathogenic Th17/Th2 cytokines, such as IL-17 and IL-13, or modulating costimulatory pathways may selectively mitigate immune-mediated podocyte injury while minimizing broader perturbations of systemic immunity (57).

Despite rapid progress, the *in vivo* spatiotemporal coordination of complement fragments, cytokine milieus, and podocyte-intrinsic checkpoints, as well as the extent to which protective circuits can be therapeutically reinforced, remains incompletely defined. Future work should prioritize discovery and validation of podocyte antigens/autoantibodies and urinary extracellular vesicle biomarkers, delineate bidirectional podocyte-immune cell crosstalk through spatial multi-omics and human genetics, and develop podocyte-targeted interventions that reduce reliance on broad immunosuppression. Together, these efforts are essential to enable precision therapies, improve long-term renal outcomes, and address unmet needs in patients with AGDs.
